# Supplementary material for: Traction Therapy for Cervical Radicular Syndrome is Statistically Significant but not Clinically Relevant for Pain Relief. A Systematic Literature Review with Meta-Analysis and Trial Sequential Analysis
Source: J Clin Med. 2020 Oct 22;9(11):3389. doi: 10.3390/jcm9113389 (PMC7690405; doi:10.3390/jcm9113389)
Supplement: Supplementary file 1 [file jcm-09-03389-s001.pdf]

Supplementary table S1: Characteristics of included studies and TIDieR.

Experimental Intervention (Cervical Traction)							
**Study, year**	**Type of traction**	**Intensity**	**Frequency**	**Setting**	**Duration**	**Who provided**	**Further information**
**Afzal, 2019[28]**	Manual Intermittent Traction	10–15% of the body weight of each patient	3 sessions per week over 3 weeks	Supine position, 25° of cervical flexion	10 minutes (10 s pull/5 s relaxation)	Experienced orthopedic manual physical therapist	/
**Fritz J.M., 2014[12]**	Mechanical Intermittent Traction	PULL: Start with 5.44 kg (12 lb) increments based on patient's tolerance, with goal of maximizing symptom reduction and centralization<br><br>RELAXATION: 50% of pull force	10 sessions over a 4-week treatment	Supine position, 15° of cervical flexion using Saunders 3D ActiveTrac or Chattanooga Triton table	15 minutes (60 s pull/20 s relaxation)	Licensed physical therapist trained by the researchers	/
**Fritz J.M., 2014[12]**	Mechanical Continuous Traction	PULL: Start with 5.44 kg (12 lb) increments based on patient's tolerance and adjusted to the maximum of 9.07 kg (20 lb)	10 sessions over a 4-week treatment	Sitting, over-door traction using a Chattanooga Overdoor Traction Device	15 minutes	Licensed physical therapist trained by the researchers	/
**Young I.A., 2009[14]**	Mechanical Intermittent Traction	Start with 9.1 kg (20 lb) or 10% of the patient's body weight (whichever was less) and increased approximately 0.91 to 2.27 kg (2–5 lb) every visit to the maximum of 15.91 kg (35 lb)	7 sessions over 4 weeks	Supine position, 15° of cervical flexion	15 minutes (50 s pull/10 s relaxation)	Physical therapist	/
**Moustafa I.M.2014[16]**	Mechanical Intermittent Traction	Start with 9.1 kg (20 lb) or 10% of the patient's body weight (whichever was less) and increased approximately 0.91 to 2.27	4 weeks	Supine position, 24° of cervical flexion	50 s pull/10 s relaxation	Therapist	/

		kg (2–5 lb) every visit to the maximum of 15.91 kg (35 lb)					
**Aydin, 2012[18]**	Mechanical Intermittent Traction	Start with 5 kg increments to the maximum of 12 Kg	15 sessions over 3 weeks	Supine position, most pain-free position of the neck	20 minutes (7 s pull/5 s relaxation)	Physical therapist	/
**Jellad, 2009[13]**	Manual Intermittent Traction	6 kg	12 sessions over 3 weeks	Supine position, most pain-free position of the neck	20 repetitions (20 s pull/10 s relaxation)	Physiotherapists	/
**Jellad, 2009[13]**	Mechanical Continuous Traction	Start with 5 kg increments to the maximum of 12 Kg	12 sessions over 3 weeks	Supine position, most pain-free position of the neck with a weightbearing pulley system	25 minutes	Physiotherapists	/
**Klaber Moffett, 1990[22]**	Mechanical Continuous Traction	From 6 to 15 lbs	12 sessions over 4 weeks	Supine position, 25° of cervical flexion	/	Physiotherapists	/

Combined and control Intervention (Other physical therapy treatments or Sham)

Study, year	Type of Intervention	Intensity	Frequency	Setting	Duration	Who provided	Further information
**Afzal, 2019[28]**	Intervertebral foramen manual opening technique	/	3 sessions per week over 3 weeks	Supine position	3 sets of 10 repetitions	Experienced orthopedic manual physical therapist	Improving rotation with one hand pulling over the restricted area
**Fritz J.M., 2014[12]**	Exercise: 1. Craniocervical flexor strengthening 2. Scapular strengthening	/	10 sessions over a 4-week treatment	1. Supine position, using an air-filled pressure sensor for feedback, then in sitting position 2. Prone horizontal abduction, side-lying forward flexion, prone extension, prone push-ups with	1. 10 seconds of contraction for 10 repetitions (increments to 30 repetitions) 2. 3 sets of 10 repetitions	Licensed physical therapist trained by the researchers	/

				emphasis on shoulder protraction			
**Young I.A., 2009[14]**	Physiotherapy treatment:<br>1. Dorsal thrust mid and high<br>2. Thoracic mobilization<br>3. Cervical mobilization<br>4. strengthening exercise<br>5. Sham traction	1. /<br>2. /<br>3. /<br>4. /<br>5. 2.27 kg (5 lb)	7 sessions over 4 weeks	1. (i.e., cervical retraction, cervical extension, deep cervical flexor strengthening, and scapular strengthening.)<br>2. (i.e., P-A glide)<br>3.(i.e., retraction, rotation, lateral glide in ULTT1, P-A glide)<br>4. (i.e., prone, supine, or sitting position)<br>5. Supine position, 15° of cervical flexion	1. /<br>2. 30 seconds or 15–20 repetitions<br>3. 30 seconds or 15–20 repetitions<br>4. /<br>5. 15 minutes (50 s pull/10 s relaxation)	Physical therapist	/
**Moustafa I.M.2014[16]**	Physiotherapy:<br>1. Laser<br>2. TENS<br>3. STM<br>4. Dorsal thrust<br>5. Strengthening	1. /<br>2. 100 Hz and a pulse duration of 125 μs<br>3. /<br>4. /<br>5. /	4 weeks	1.Forward lean sitting position<br>2. Prone position<br>3. Involved upper extremity positioned in abduction and external rotation<br>4. A/P force in supine or distraction force in sitting position<br>5. Deep cervical flexors, shoulder retractors, serratus anterior	1. /<br>2.20 minutes<br>3. /<br>4. /<br>5. Holding contraction	Therapist	Manual pressure was applied to the soft tissues of the upper quadrant in a deep, stroking manner concentrated on any tissues that were graded as tight or tender.
**Aydin, 2012[18]**	Physiotherapy:<br>1. US<br>2. hot packs<br>3. TENS<br>4. Isometric	1. 1 watt/cm2<br>2. /<br>3. 60 Hz and impulse duration of 100 μ/sec	15 sessions over 3 weeks	1. /<br>2. /<br>3. /<br>4. /<br>5. /	1.10 minutes<br>2. 20 minutes<br>3. 20 minutes<br>4. 3 sets of 10	Physical therapist	/

	cervical<br>strengthening<br>5. Stretching	4. /<br>5. /			repetitions<br>5. /		
**Jellad, 2009[13]**	Physiotherapy:<br>1. US<br>2. Laser<br>3. Massage<br>4. Cervical spine mobilization<br>5. Isometric cervical strengthening<br>6. Stretching	1. /<br>2. /<br>3. /<br>4. /<br>5. /<br>6.	12 sessions over 3 weeks	1. /<br>2. /<br>3. /<br>4. /<br>5. /<br>6.	1. /<br>2. /<br>3. /<br>4. /<br>5. /<br>6.	Physiotherapists	/
**Klaber Moffett, 1990[22]**	Sham traction	2 lbs	12 sessions over 4 weeks	Supine position, 25° of cervical flexion	/	Physiotherapists	/

P-A= posterior to anterior; STM= Soft tissue mobilization; TENS= Transcutaneous electrical nerve stimulation; ULTT1= Upper Limb Tension Test 1; US= Ultrasound.

Supplementary Table S2: Synthesis of Evidence of traction compared to Sham traction for radicular syndrome.

Traction compared to sham traction for radicular syndrome					
**Patient or population**: Radicular syndrome<br>**Intervention**: Traction<br>**Comparison**: Sham traction					
**Outcomes**	**No of participants (studies) Follow up**	**Certainty of the evidence (GRADE)**	**Relative effect (95% CI)**	**Anticipated absolute effects**	
				**Risk with sham traction**	**Risk difference with traction***
Pain Intensity assessed with: VAS scale 0–100	100 (1 RCT)	⨁⨁◯◯ LOW [a,b]	-	The mean pain was **36.7** pain intensity	MD **5 pain intensity lower** (14.98 lower to 4.98 higher)

***The risk in the intervention group** (and its 95% confidence interval) is based on the assumed risk in the comparison group and the **relative effect** of the intervention (and its 95% CI).

CI: Confidence interval; **MD:** Mean difference

GRADE Working Group grades of evidence
High certainty: We are very confident that the true effect lies close to the estimate of the effect.
Moderate certainty: We are moderately confident in the effect estimate. The true effect is likely to be close to the estimate of the effect, but there is a possibility that it is substantially different.
Low certainty: Our confidence in the effect estimate is limited. The true effect may be substantially different from the estimate of the effect.
Very low certainty: We have very little confidence in the effect estimate. The true effect is likely to be substantially different from the estimate of effect .

Explanations: a. Downgraded (-2) for risk of bias. Selection bias (unclear random sequence generation and allocation). b. Downgraded (-1) for imprecision. OIS is not met, sample size was small, with wide 95% CI including effect estimates that are clinically important and unimportant.

Supplementary Table S3: Synthesis of Evidence of Overall Analysis.

The quality of evidence starting from "high" was lowered by one level for each factor that was not met. For instance, adopting the estimate of DARIS in TSA, we downgraded for distortion in the imprecision domain if the OIS was not met or if it had been met and CI overlapped no effect, and the upper and lower confidence limits crossed the MID in 20 out of 100 in either direction, thus failing to exclude important benefits or important harms (imprecision). Abbreviations: CI=Confidence intervals; DARIS=; MID= Minimal important difference; OIS= optimal information size; TSA=Trial sequential analysis.

Traction + Other treatments compared versus other treatments individually for radicular syndrome

Patient or population: Radicular syndrome
Intervention: Traction + other treatments
Comparison: Other treatments

Outcomes	№ of participants (studies) Follow up	Certainty of the evidence (GRADE)	Relative effect (95% CI)	Anticipated absolute effects	
				**Risk with other treatments**	**Risk difference with traction + other treatments***
Pain Intensity assessed with: VAS scale 0–100	405 (6 RCTs)	⊕⊕○○ LOW [a]	-	The mean pain Intensity was –**5** to **93** pain intensity	MD **5.93 pain intensity lower** (11.81 lower to 0.04 lower)

***The risk in the intervention group** (and its 95% confidence interval) is based on the assumed risk in the comparison group and the **relative effect** of the intervention (and its 95% CI).

CI: Confidence interval; **MD:** Mean difference

GRADE Working Group grades of evidence
High certainty: We are very confident that the true effect lies close to the estimate of the effect.
Moderate certainty: We are moderately confident in the effect estimate. The true effect is likely to be close to the estimate of the effect, but there is a possibility that it is substantially different.
Low certainty: Our confidence in the effect estimate is limited. The true effect may be substantially different from the estimate of the effect.
Very low certainty: We have very little confidence in the effect estimate. The true effect is likely to be substantially different from the estimate of effect.

Explanations: a. Downgraded (-2) for risk of bias (detection and performance bias).

Supplementary Table S4: Synthesis of Evidence of Subgroup Analysis (a).

Traction + other treatments versus other treatments individually for radicular syndrome					
**Patient or population**: Radicular syndrome<br>**Subgroup**: Mechanical and manual<br>**Intervention**: Traction + other treatments<br>**Comparison**: Other treatments					
**Outcomes**	**№ of participants (studies) Follow up**	**Certainty of the evidence (GRADE)**	**Relative effect (95% CI)**	**Anticipated absolute effects**	
				**Risk with other treatments**	**Risk difference with traction + other treatments***
Pain Intensity (Mechanical Traction) assessed with: VAS 0–100	357 (5 RCTs)	⊕⊕◯◯ LOW [a]	-	The mean pain intensity ranged from **26–63** pain intensity	MD **6.21 pain intensity lower** (11.69 lower to 0.73 lower)
Pain Intensity (Manual Traction) assessed with: VAS 0–100	48 (2 RCTs)	⊕◯◯◯ VERY LOW [a,b,c]	-	The mean pain intensity ranged from **25–63** pain intensity	MD **9.26 pain intensity lower** (38.54 lower to 20.03 higher)

***The risk in the intervention group** (and its 95% confidence interval) is based on the assumed risk in the comparison group and the **relative effect** of the intervention (and its 95% CI).

CI: Confidence interval; **MD:** Mean difference

GRADE Working Group grades of evidence
High certainty: We are very confident that the true effect lies close to the estimate of the effect.
Moderate certainty: We are moderately confident in the effect estimate. The true effect is likely to be close to the estimate of the effect, but there is a possibility that it is substantially different.
Low certainty: Our confidence in the effect estimate is limited. The true effect may be substantially different from the estimate of the effect.
Very low certainty: We have very little confidence in the effect estimate. The true effect is likely to be substantially different from the estimate of effect.

Explanations: a. Downgraded (-2) for risk of bias (detection and performance bias); b. Downgraded (-1) for inconsistency. I2 > 75%; c. Downgraded (-1) for imprecision. Small sample size with wide 95% CI including effect estimates that are clinically important and unimportant.

Supplementary table S5: Synthesis of Evidence of subgroup (b).

Traction + other treatments versus other treatments invidually for radicular syndrome					
**Patient or population**: adicular syndrome<br>**Subgroup**: Continuous and intermittent<br>**Intervention**: Traction + other treatments<br>**Comparison**: Other treatments					
**Outcomes**	**№ of participants (studies) Follow up**	**Certainty of the evidence (GRADE)**	**Relative effect (95% CI)**	**Anticipated absolute effects**	
				**Risk with other treatments**	**Risk difference with traction + other treatments***
Pain Intensity (Continuous Traction) assessed with: VAS 0–100	60 (2 RCTs)	⨁◯◯◯ VERY LOW [a,b]	-	The mean pain Intensity ranged from **26–53** pain intensity	MD **13.08 pain intensity lower** (24.29 lower to 1.88 lower)
Pain Intensity (Intermittent Traction) assessed with: VAS 0–100	345 (6 RCTs)	⨁◯◯◯ VERY LOW [a,c]	-	The mean pain Intensity ranged from **25–63** pain intensity	MD **4.27 pain intensity lower** (10.67 lower to 2.12 higher)

***The risk in the intervention group** (and its 95% confidence interval) is based on the assumed risk in the comparison group and the **relative effect** of the intervention (and its 95% CI).

CI: Confidence interval; **MD:** Mean difference

GRADE Working Group grades of evidence
High certainty: We are very confident that the true effect lies close to the estimate of the effect.
Moderate certainty: We are moderately confident in the effect estimate. The true effect is likely to be close to the estimate of the effect, but there is a possibility that it is substantially different.
Low certainty: Our confidence in the effect estimate is limited. The true effect may be substantially different from the estimate of the effect.
Very low certainty: We have very little confidence in the effect estimate. The true effect is likely to be substantially different from the estimate of effect.

Explanations: a. Downgraded (-2) for risk of bias (detection and performance bias); b. Downgraded (-1) for imprecision. OIS is not met, sample size was small; c. Downgraded (-1) for imprecision. Wide 95% CI including effect estimates that are clinically important and unimportant.
